# Multiple Pituitary Hormone Deficiency Due to Gunshot Injury in a 6-Year-Old Girl

**DOI:** 10.4274/Jcrpe.1027

**Published:** 2013-09-18

**Authors:** Hüseyin Demirbilek, Mehmet Nuri Özbek, Rıza Taner Baran, Ahmet Baran

**Affiliations:** 1 Diyarbakır Children’s State Hospital, Pediatric Endo crinology, Diyarbakır, Turkey; 2 Diyarbakır Children’s State Hospital, Radiology, Diyarbakır, Turkey

## Abstract

Gunshot injuries (GSI) of the cranial area have an extremely high mortality rate. Herein, we present a girl who has been living with a bullet in the posterior sellar region. A 6-year-old girl was admitted with complaints of headache, polyuria and polydypsia, which started after a GSI. At the time of admission the patient’s anthropometric, physical and neurological examinations were normal. Urine output was 5.5 L/m2/24h. A water deprivation test suggested central diabetes insipidus, which responded to treatment. Evaluation of other pituitary hormones revealed central hypothyroidism and growth hormone deficiency. Pituitary hormone deficiency must be kept in mind in patients injured by a gunshot to the sellar/parasellar region.

**Conflict of interest:**None declared.

## INTRODUCTION

Gunshot injuries (GSI) to the cranial area have an extremely high mortality rate ranging from 51% to 84% (1,2,3,4,5). The most important prognostic factors affecting mortality rate are the course of the bullet and the Glasgow Coma Scale (GCS) score at the time of initial evaluation (6). Respiratory and circulatory status at presentation, the diameter and reactivity of the pupils, and the presence or absence of coagulopathy constitute the other prognostic factors. Intraventricular haemorrhage suggests a poor prognosis. The most important cause of death is hernia associated with an increased intracranial pressure (1,7,8). On the other hand, post-traumatic complications in individuals who survive GSI constitute a considerable health problem. Pituitary hormone deficiency after GSI to the cranial area has been reported in a limited number of cases, mainly in adults (8,9,10,11,12). Herein, we present a 6-year-old girl who suffered a GSI and is living with a bullet in the posterior sellar region which causes multiple pituitary hormone deficiency. 

## CASE REPORT

**Figure 1:**
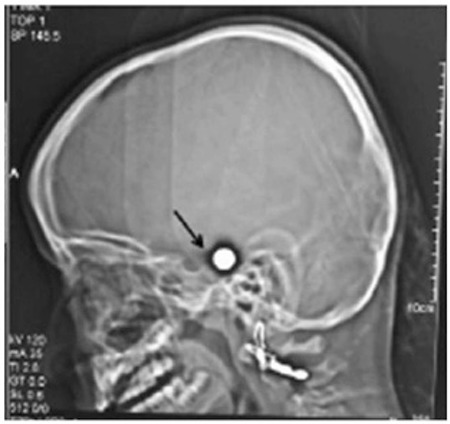
Bullet located in the posterior wall of the sella

 A 6-year-old girl was admitted to our clinic with complaints of headache, polyuria, and polydypsia. The patient was reported to be healthy until 5 months ago, when she suffered a head injury with an unknown foreign object while playing in the garden. Radiological examination revealed a bullet in her skull thought to be a GSI (Figure 1). Since the bullet was adjacent to vital regions of the brain, she was deemed inoperable, and was left to live with a bullet in her brain. Her complaints, mainly consisting of headache, polyuria and polydypsia, started at that time. Her family history was unremarkable. At the time of admission, her height was 113.5 cm (25th-50th percentile) and weight was 21 kg (50th percentile). Other physical findings were normal.

Urine density at presentation was 1001. Urine output was 4.5 L/24h (5.5 L/m2/24h). A water deprivation test showed failure to concentrate urine (Table 1). After a test dose of nasal desmopressin (5μg), urine output decreased and urine osmolarity increased. She was diagnosed as a case of central diabetes insipidus. The complaints resolved with a single 5μg daily maintenance dose of intranasal desmopressin acetate.

At follow-up, hormonal evaluation revealed central hypothyroidism (Table 2). A euthyroid state was achieved using Na-L-thyroxine therapy. Baseline morning cortisol was 7.7 µg/dL and low-dose adrenocorticotropic hormone (ACTH) test showed normal adrenal function (Table 2). A very low growth rate (0.2 cm/6 months), detected at follow-up, suggested growth hormone (GH) deficiency. GH response to L-dopa and clonidine stimulation tests was inadequate, and recombinant human GH therapy was started. With this treatment, except for a slight headache, the patient has no complaints and appears healthy.

## DISCUSSION

GSIs to the cranial region, which are commonly caused by celebratory gun shooting, have a very high mortality rate. Although this injury often results in death, in some cases the injured person can survive. In this case, the localization of the bullet plays a considerable role in the prediction of the prognosis and post-injury complications. A bullet located in non-vital regions of the brain can be removed successfully by surgery. However, in cases with bullet settled in vital areas, to avoid further damage from surgery, the bullet may be left in the cranium. In this instance, according to the location of the bullet, various complications such as headache, visual disorders, and infection may be inevitable. Post-traumatic hypopituitarism has been reported in patients suffering from traumatic brain injury (13,14,15,16). While it is extremely rare in the paediatric age group, multiple pituitary hormone deficiency due to cranial gunshot injury has been reported (8,9,10,11,12). Central diabetes insipidus is the mostly commonly reported neuroendocrine dysfunction of traumatic brain injury. Other and less frequent outcomes of traumatic brain injury are GH, thyroid stimulating hormone, ACTH, follicle-stimulating hormone, and luteinizing hormone deficiencies (12,17).

We did not have the means to perform pituitary or cranial MR imaging in our patient, thus, we were not able to completely rule out other causes of hypopituitarism. However, evidence such as the patient being healthy until suffering the cranial injury and the onset of the symptoms following the injury strongly suggest that the multiple pituitary hormone deficiency was caused by GSI. Moreover, at the time of admission, the patient’s height was between the 25th and 50th percentiles for age and sex, but during follow-up, growth velocity was found to be extremely low, and a sharp decline was observed in the growth curve. The inadequate GH response to the GH stimulation test performed with L-dopa and clonidine also supported the presence of an acquired pituitary dysfunction.

Since the patient was pre-pubertal, we could not evaluate her gonadotropins and gonadal functions. Although her ACTH level was within the normal range, the risk of development of ACTH and gonadotropin deficiency may exist and the hormonal profile needs to be monitored at regular intervals.

In conclusion, the findings of this patient demonstrate that GSI can cause multiple pituitary hormone deficiency. Although it is extremely rare, pituitary hormone deficiency must be kept in mind in patients presenting with GSIs and living with a bullet in their brain, especially in the sellar/parasellar regions. 

## Figures and Tables

**Table 1:**
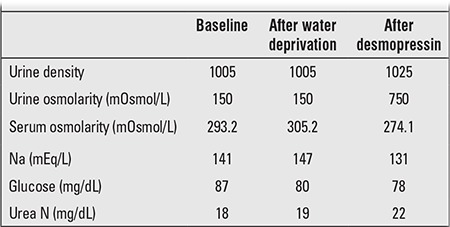
Results of the water deprivation test*

**Table 2:**
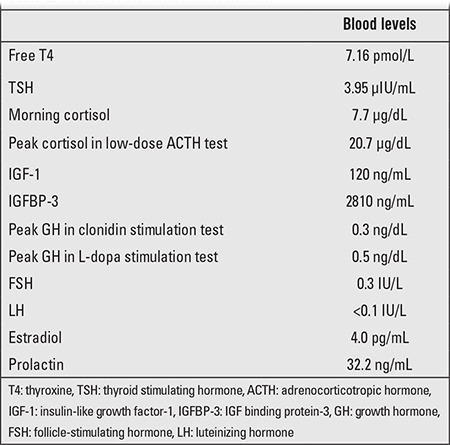
Results of the hormonal evaluation
